# Supplementary material for: Occurrence and multilocus genotyping of Giardia duodenalis in captive non-human primates from 12 zoos in China
Source: PLoS One. 2020 Feb 4;15(2):e0228673. doi: 10.1371/journal.pone.0228673 (PMC6999901; doi:10.1371/journal.pone.0228673)
Supplement: S1 Table — (DOC) [file pone.0228673.s001.doc]

**S1 Table: Occurrence of *Giardia duodenalis* in different species of nonhuman primates.**

| Common name | Host location | No. of specimens tested | No. (%) of positive specimens |
| --- | --- | --- | --- |
| Golden monkey | Shaanxi Rare and Wildlife Zoo (26),  Chengdu Zoo (18), Guiyang Forest Wildlife Zoo (15),  Nanjing Zoo (3), Kunming Zoo (2),  Chongqing Zoo (2), Yangzhou Zoo (1),  Dalian Zoo (1), Guangzhou Zoo (1) | 69 | 12 (17.39) |
| Nagao monkey | Chongqing Zoo (4), Dalian Zoo (3), Chengdu Zoo (2), Beijing Zoo (2), Kunming Zoo (1) | 12 | 0 |
| The green monkey | Changsha Zoo (4), Dalian Zoo (2), Chengdu Zoo (1) | 7 | 0 |
| Phayre's leaf monkey | Kunming Zoo (1) | 1 | 0 |
| Squirrel monkey | Guiyang Forest Wildlife Zoo (15), Changsha Zoo (6),  Chengdu Zoo (3), Chongqing Zoo (1),  Kunming Zoo (1), Yangzhou Zoo (1),  Dalian Zoo (1), Suzhou Zoo (1),  Guangzhou Zoo (1) | 30 | 1 (3.33) |
| Flat-topped monkey | Changsha Zoo (3) | 3 | 0 |
| Francois' leaf monkey | Chengdu Zoo (1), Dalian Zoo (1),  Yangzhou Zoo (1), Kunming Zoo (1),  Chongqing Zoo (1), Guangzhou Zoo (1) | 6 | 0 |
| Hussar monkey | Guiyang Forest Wildlife Zoo (6), Changsha Zoo (4),  Chongqing Zoo (2), Kunming Zoo (1), Dalian Zoo (1),  Suzhou Zoo (1), Guangzhou Zoo (1) | 16 | 0 |
| Black spider monkey | Kunming Zoo (1), Beijing Zoo (2) | 3 | 0 |
| Mandrill | Chengdu Zoo (3), Guiyang Forest Wildlife Zoo (3),  Changsha Zoo (1), Chongqing Zoo (1),  Kunming Zoo (1), Dalian Zoo (1), Suzhou Zoo (1) | 11 | 0 |
| Rhesus macaque | Nanjing Zoo (10), Chongqing Zoo (7),  Chengdu Zoo (4), Yangzhou Zoo (1), Dalian Zoo (1) | 23 | 0 |
| Pig-tailed macaque | Chongqing Zoo (1) | 1 | 0 |
| Crab-eating macaque | Changsha Zoo (3), Chengdu Zoo (1) | 4 | 0 |
| Japanese macaques | Dalian Zoo (1), Chengdu Zoo (1), Suzhou Zoo (1) | 3 | 1 (33.33 ) |
| Black crown macaques | Beijing Zoo (2) | 2 | 0 |
| Tibetan macaque | Chengdu Zoo (1) | 1 | 0 |
| Lion-tailed macaque | Chongqing Zoo (1), Dalian Zoo (1) | 2 | 0 |
| Baboons | Guiyang Forest Wildlife Zoo (5), Changsha Zoo (3),  Chongqing Zoo (3), Beijing Zoo (3), Yangzhou Zoo (2), Dalian Zoo (2), Suzhou Zoo (2), Chengdu Zoo (2),  Kunming Zoo (1) | 23 | 1 (4.35) |
| Africa black-and-white colobus | Suzhou Zoo (1) | 1 | 1 (100) |
| Colobus | Changsha Zoo (3), Kunming Zoo (1),  Guangzhou Zoo (1) | 5 | 0 |
| Eastern african back-white colobus | Dalian Zoo (1) | 1 | 0 |
| White-cheeked gibbon | Guiyang Forest Wildlife Zoo (4), Changsha Zoo (3),  Chongqing Zoo (3), Chengdu Zoo (1), Beijing Zoo (1),  Guangzhou Zoo (1), Nanjing Zoo (1) | 14 | 1 (7.14) |
| White-browed gibbon | Guiyang Forest Wildlife Zoo (10), Nanjing Zoo (1),  Chongqing Zoo (1), Chengdu Zoo (1) | 13 | 0 |
| The yellow-cheeked gibbon | Nanjing Zoo (1) | 1 | 0 |
| Gibbons | Kunming Zoo (2), Beijing Zoo(2), Dalian Zoo (1),  Yangzhou Zoo (1), Guangzhou Zoo (1) | 7 | 0 |
| Red gorilla | Chengdu Zoo (1), Chongqing Zoo (1) | 2 | 0 |
| Chimpanzee | Changsha Zoo (3), Dalian Zoo (3), Suzhou Zoo (1),  Chengdu Zoo (1), Guangzhou Zoo (1) | 9 | 2 (22.22) |
| Common chimpanzee | Chongqing Zoo (1) | 1 | 0 |
| Ring-tailed lemur | Changsha Zoo (6), Kunming Zoo (2),  Yangzhou Zoo (2), Chongqing Zoo (2),  Chengdu Zoo (2), Dalian Zoo (1), Suzhou Zoo (1) | 16 | 5(31.25) |
| Ruffed lemur | Dalian Zoo (1), Suzhou Zoo (1) | 2 | 1(50.00) |
| Weeper capuchin | Chongqing Zoo (1), Dalian Zoo (1) | 2 | 0 |
| Black-capped capuchin | Changsha Zoo (4), Chengdu Zoo (4),  Chongqing Zoo (1), Kunming Zoo (1), Dalian Zoo (1) | 11 | 0 |
| Total：32 primate species | Eight provinces and two municipalities | 302 | 25(8.28) |
