# Supplementary material for: Occurrence and multilocus genotyping of Giardia duodenalis in captive non-human primates from 12 zoos in China
Source: PLoS One. 2020 Feb 4;15(2):e0228673. doi: 10.1371/journal.pone.0228673 (PMC6999901; doi:10.1371/journal.pone.0228673)
Supplement: S2 Table — (DOCX) [file pone.0228673.s002.docx]

**S2 Table.** **Variations in *bg*, *tpi* and *gdh* nucleotide sequences among the subtypes of *Giardia duodenalis* assemblage B from NHPs**

| **Genetype** | **Sub-assemblages** | **GenBank accession number** | **Nucleotide at position** | | | | | | | | | | | | | | |
| --- | --- | --- | --- | --- | --- | --- | --- | --- | --- | --- | --- | --- | --- | --- | --- | --- | --- |
|  |  |  | **77** | **196** | **201** | **230** | **231** | **233** | **249** | **319** | **320** | **331** | **332** | **417** | **473** | **490** | **496** |
| *bg* | BIII**(Ref.)** | KU504724 | A | C | G | C | A | A | C | G | C | C | T | G | A | A | G |
|  | BIII-1(DLGB04) | MK909127^#^ | • | • | • | • | • | • | T | • | • | • | • | A | • | • | • |
| *tpi* | MB10**(Ref.)** | KJ888988 | C | A | A | G | G | G | G | G | T | G | G | A | G | C | A |
|  | MB10-1(DLGT04) | MK909131**^#^** | • | • | • | • | A | • | • | • | • | • | • | • | • | • | • |
|  | B14**(Ref.)** | KF679737 | C | G | A | G | A | A | G | T | A | G | C | A | G | C | A |
|  | B14-1(GYGT26,GYGT28) | MK909136**^#^** | T | A | • | • | • | • | • | • | • | • | • | • | • | T | G |
|  | WB8**(Ref.)** | KF679738 | C | C | A | C | G | C | G | C | T | A | G | A | G | C | A |
|  | WB8-1(GYGT23,GYGT55) | MK909135**^#^** | • | • | • | • | • | • | • | • | • | • | A | • | • | • | • |
|  | MB9**(Ref.)** | KJ888985 | C | C | A | G | G | C | G | A | G | G | A | G | T | G | T |
|  | MB9-1(SZGT10) | MK952610**^#^** | • | • | • | A | • | • | • | G | • | • | • | • | • | • | • |
| *gdh* | DN7**(Ref.)** | MG746610 | T | T | G | A | A | C | C | T | C | A | C | C | G | A | A |
|  | DN7-1(YZGG05, YZGG06) | MK952598**^#^** | • | • | • | • | • | • | • | • | • | • | • | • | A | • | • |
|  | BIV**(Ref.)** | KF679731 | T | G | G | G | C | T | A | T | C | T | A | G | C | A | G |
|  | BIV-1(GYGG97) | MK952606**^#^** | • | • | • | • | • | • | • | • | • | C | • | • | • | • | • |

Note: “^#^”: GenBank accession number in this study； “•”: represent the same base.
