# Supplementary material for: Occurrence and multilocus genotyping of Giardia duodenalis in captive non-human primates from 12 zoos in China
Source: PLoS One. 2020 Feb 4;15(2):e0228673. doi: 10.1371/journal.pone.0228673 (PMC6999901; doi:10.1371/journal.pone.0228673)
Supplement: Attachment — Submitted filename: Response to Reviewers.docx [file pone.0228673.s004.docx]

Dear Editor:

Thank you very much for your letter and the reviewers’ comments regarding our manuscript submitted to “PLoS One” (Manuscript Number: PONE-D-19-24653R1). We have checked the article and revised it according to the comments, and carefully proof-read the manuscript errors.

We hope, with these modifications and improvements based on your suggestion and the reviewer’s comments, the quality of our manuscript would meet the publication standard of “PLoS One”. Once again, we acknowledge your comments and constructive suggestions very much, which are valuable in improving the quality of our manuscript.

We submit here the revised manuscript as well as a list of changes. Thanks again for your reconsideration of our manuscript for publication in your journal. If you have any question about this paper, please don’t hesitate to let me know.

Best wishes for you!

Sincerely yours,

Zhijun Zhong

College of Veterinary Medicine

Key Laboratory of Animal Disease and Human Health of Sichuan Province

Sichuan Agricultural University

Chengdu 611130, P. R. China

E-mail: zhongzhijun488@126.com

**Response to Reviewer:**

Thank you to the reviewers for their time and thoughtful comments, many of which have been incorporated into the revised manuscript. This revision was carried out according to the suggestions of the reviewers. Detailed comments are as follows.

**Reviewer #1:**

**Major**

1. Lines 78-91: In my initial appraisal, I requested information about how zoological gardens were selected, approached, and invited to participate in the study. I also requested information regarding the inclusion/exclusion criteria for selecting samples, and the presence/absence of other parasitic, viral, and bacterial infections. None of these have been addressed in the revised version of the manuscript. Amend.

Answer: Thanks for your comments. Some related description are add in both Intrduction and Sample collection section, and highlighted them in the revised tracked-manuscript. (line 74-81, line 90-92 and line 95-98)

To date, most Chinese studies evaluating *G. duodenalis* infection in NHPs have focused on a single zoo or localized area. In this project, ongoing epidemiological surveys on intestinal zoonotic parasites of *G. duodenalis*, expanded previous studies to large-scale investigation of zoos and NHP species in China. As our last replied in the MS R1 version, in this study we only get 12 zoos permission to collect samples. The 12 zoos were distributed in seven different geographical divisions [Central China: Hunan province (Changsha Zoo); North China: Beijing (Beijing Zoo); East China: Jiangsu province ( Suzhou Zoo, Yangzhou Zoo and Nanjing Zoo); Southern China: Guangdong province (Guangzhou Zoo); Northwestern China: Shaanxi (Shaanxi Rare and Wildlife Zoo); Northeastern China: Liaoning province (Dalian Zoo); Southwest China: Sichuan province (Chengdu Zoo), Chongqing (Chongqing Zoo), Guizhou province (Guiyang Forest Wildlife Zoo), Yunnan province (Kunming Zoo)]. Our present project evaluated 302 NHP fecal samples (including 32 primate species) from 12 zoos distributed across eight Chinese provinces and two municipalities (Chongqing and Beijing), to better understand *G. duodenalis* infection in captive NHPs throughout China. In this project, we only focus on the occurrence of *G. duodenalis* infections in non-human primates, the presence/absence of other parasitic, viral, and bacterial infections is unknown. The 12 zoos have adequate facilities to accommodate the different species of primates in indoor enclosure, different species live on separated places. The 12 zoos are distributed throughout China (Fig 1), and the feed managements are all according to the Standard Rule of Chinese Association of Zoological Gardens.

2. Lines 149-151: please note that this statement does not fully clarify the initial issue. Please clearly state here that PCR protocols based on single-copy genes (e.g. *gdh*, *bg*, and *tpi*) had considerable lower diagnostic sensitivities than those based on multiple-copy genes (e.g. *ssu*). In practical terms, this means that infection rates reported in the present study are an underestimation of the true ones. Amend.

Answer: Thanks for your comments. We added relevant description and highlighted it in the revised manuscript. (lines 162-164, lines 166-167)

3. Sequence analyses: please clearly state in the text that sequences with ambiguous positions (double peaks) were not included in the phylogenetic analyses.

Answer: Thanks for your comments. We added relevant description and highlighted it in the revised manuscript. (lines 127-128)
